# Enzymatic Active Release of Violacein Present in Nanostructured Lipid Carrier by Lipase Encapsulated in 3D-Bioprinted Chitosan-Hydroxypropyl Methylcellulose Matrix With Anticancer Activity

**DOI:** 10.3389/fchem.2022.914126

**Published:** 2022-07-07

**Authors:** Ignacio Rivero Berti, Boris E. Rodenak-Kladniew, Sergio F. Katz, Eva Carolina Arrua, Vera A. Alvarez, Nelson Duran, Guillermo R. Castro

**Affiliations:** ^1^ Laboratorio de Nanobiomateriale, CINDEFI, Departamento de Química, Facultad de Ciencias Exactas, CONICET (CCT La Plata), Universidad Nacional de La Plata (UNLP), La Plata, Argentina; ^2^ Instituto de Investigaciones Bioquímicas de La Plata (INIBIOLP), CONICET-UNLP, CCT-La Plata, Facultad de Ciencias Médicas, La Plata, Argentina; ^3^ Max Planck Laboratory for Structural Biology, Chemistry and Molecular Biophysics of Rosario (MPLbioR, UNR-MPIbpC), Partner Laboratory of the Max Planck Institute for Biophysical Chemistry (MPIbpC, MPG), Centro de Estudios Interdisciplinarios (CEI), Universidad Nacional de Rosario, Rosario, Argentina; ^4^ Centro de Investigación y Desarrollo en Materiales Avanzados y Almacenamiento de Energía de Jujuy-Univ. Nac., de Jujuy, Argentina; ^5^ Grupo de Materiales Compuestos Termoplásticos (CoMP), Instituto de Investigaciones en Ciencia y Tecnología de Materiales (INTEMA), Facultad de Ingeniería, Universidad Nacional de Mar del Plata (UNMDP), CONICET, Mar del Plata, Argentina; ^6^ Laboratory of Urogenital Carcinogenesis and Immunotherapy, Department of Structural and Functional Biology, Universidade Estadual de Campinas (UNICAMP), Campinas, Brazil; ^7^ Nanomedicine Research Unit (Nanomed), Center for Natural and Human Sciences (CCNH), Universidade Federal do ABC (UFABC), Santo André, Brazil

**Keywords:** Violacein, nanostructured lipid carriers, Lipase, chitosan, 3D-bioprinter, controlled release, Violacein active release

## Abstract

Violacein (Viol) is a bacterial purple water-insoluble pigment synthesized by *Chromobacterium violaceum* and other microorganisms that display many beneficial therapeutic properties including anticancer activity. Viol was produced, purified in our laboratory, and encapsulated in a nanostructured lipid carrier (NLC). The NLC is composed of the solid lipid myristyl myristate, an oily lipid mixture composed of capric and caprylic acids, and the surfactant poloxamer P188. Dormant lipase from *Rhizomucor miehei* was incorporated into the NLC-Viol to develop an active release system. The NLC particle size determined by dynamic light scattering brings around 150 nm particle size and *ζ*≈ −9.0 mV with or without lipase, but the incorporation of lipase increase the PdI from 0.241 to 0.319 (≈32%). For scaffold development, a 2.5 hydroxypropyl methylcellulose/chitosan ratio was obtained after optimization of a composite for extrusion in a 3D-bioprinter developed and constructed in our laboratory. Final Viol encapsulation efficiency in the printings was over 90%. Kinetic release of the biodye at pH = 7.4 from the mesh containing NLC-lipase showed roughly 20% Viol fast release than without the enzyme. However, both Viol kinetic releases displayed similar profiles at pH = 5.0, where the lipase is inactive. The kinetic release of Viol from the NLC-matrices was modeled and the best correlation was found with the Korsmeyer-Peppas model (R^2^ = 0.95) with *n* < 0.5 suggesting a Fickian release of Viol from the matrices. Scanning Electron Microscope (SEM) images of the NLC-meshes showed significant differences before and after Viol’s release. Also, the presence of lipase dramatically increased the gaps in the interchain mesh. XRD and Fourier Transform Infrared (FTIR) analyses of the NLC-meshes showed a decrease in the crystalline structure of the composites with the incorporation of the NLC, and the decrease of myristyl myristate in the mesh can be attributed to the lipase activity. TGA profiles of the NLC-meshes showed high thermal stability than the individual components. Cytotoxic studies in A549 and HCT-116 cancer cell lines revealed high anticancer activity of the matrix mediated by mucoadhesive chitosan, plus the biological synergistic activities of violacein and lipase.

## 1 Introduction

Bacterial metabolites make up a great pool of molecules that, in addition to having a wide range of biological activities, are relatively simple and inexpensive to produce in a sustainable way (Venil et al., 2020). In 2020, the GLOBALCAN reported more than 19 million new cases and 10 million deaths. Additionally, 80% of new cases occur in individuals over 50 years of age, so as the world population ages, the prevalence of these diseases grows accordingly (Ferlay et al., 2020). Violacein (Viol) is a violet-purple pigment synthesized by numerous Gram-negative bacterial strains that have demonstrated beneficial antibacterial, antifungal, trypanocide, antiviral, and antitumoral activities, among others (Durán et al., 2021). Particularly, Viol inhibits the expression of some cell markers associated with proliferation (e.g., cyclin-dependent, mitogen-activated kinases, etc.), inhibit metalloproteinases, enzymes essential to metastatic processes, and induces tumoral cell death by different mechanisms mentioned recently (Durán et al., 2016).

Viol is practically insoluble in aqueous media and its high hydrophobicity correlated well with the parameter XLogP3-aa = 2.7. XLogP3-aa is a statistical parameter used to estimate the hydrophobicity/hydrophilicity of a molecule considering the contribution of each atom, hydrogen bridges, and the terminal groups (Cheng et al., 2007). The Viol insolubility in aqueous media is a serious limitation for its prospective application as a therapeutic agent because of low bioavailability. Besides, it is estimated that between 50% and 90% of the new drugs in development and 40% of the drugs currently available in the market present the same drawback (Liu, 2018). Thus, Viol could be a promising new drug but also an excellent therapeutic agent if a suitable drug delivery system is developed. Since Viol is a hydrophobic molecule, lipid systems can be considered as a potential best drug carrier (Rivero Berti et al., 2020). Among them, liposomes, and lipid nanoparticles are probably the most popular structures employed for the development of drug delivery systems because of their simple preparation, non-toxic, easy scale-up, and reproducibility. However, liposomes are thermodynamically unstable, with high structural dependence on environmental conditions, unknown cargo concentration, and unpredictable kinetic release. Meanwhile, solid lipid nanoparticles display several advantages, such as improving the solubility of poorly soluble drugs, and also increasing circulation time, thus avoiding possible resistance mechanisms displayed by tumor cells, such as efflux pumps (Creixell and Peppas, 2012). Solid lipid delivery systems propose platforms that are relatively simple to prepare, extremely stable, have high loading efficiency for hydrophobic drugs and have high biocompatibility (Scioli Montoto et al., 2020). Although SLNs have a high encapsulation efficiency, in some cases when the nanoparticles mature, the lipids of the matrix crystallize, and the drug is expelled during the process with the consequent loss of loaded drug. In nanostructured lipid carriers (NLC) systems an oily lipid is added to the solid lipid core to introduce entropy and prevent and/or reduce lipid crystallization (Gordillo-Galeano and Mora-Huertas, 2018). In the present work, myristyl myristate (MM) was selected as the main lipid component for the NLC, since MM has shown advantages in NLC preparation such as emulsification enhancer, effective thickener, low acute toxicity in oral or dermal tests, and its extensively used in personal care and cosmetic products (Islan et al., 2016; Shilling, 1990; Berti, et al., 2019). Also, a commercial mixture of caprylic and capric acids was employed in the NLC preparation because it is an oily liquid lipid at RT and used as a thickener to improve the texture and stability of emulsions, it is biodegradable and commonly used in pharmaceutical and cosmetic formulations (Islan et al., 2016).

However, one of the main problems in cancer therapies for the delivery of nanoparticles containing hydrophobic and/or environmentally sensitive drugs is the low attachment of nanoparticles to the cancer cell surfaces, insufficient drug penetration, and inadequate drug release kinetics (Wang et al., 2018). The consequent drug release failure can result in molecular degradation under physiological conditions (i.e., lytic enzymes), fast clearance, and/or formation of insoluble complexes (i.e., π-stacking) that result in low drug bioavailability and could produce also undesirable side effects. A potential solution could be the development of a stimuli-responsive controlled release system (SRCRS), which will be particularly useful for the treatment of solid tumors. Among the main advantages of SRCRS is the circumvention of premature drug clearance, high accumulation on targeted cells or tissues, and proper stimuli-responsive drug release with controlled kinetics (Rahim et al., 2021). Importantly, SRCRS reduces the drug amount to be administered and gets the same therapeutic results, which is a substantial advantage for improving the individuals’ quality of life, and principally for oncological patients. The use of enzymes for the development of SRCRS to release the cargo in place and with appropriate kinetics was described. Enzymes such as proteases, glycosidases, and lipases were tested for the release of growth factors (i.e., hepatocyte growth factors and VEGF), insulin, doxorubicin, nitric oxide, etc. from nanoparticle and hydrogel devices (Chandrawati, 2016; Wang et al., 2018; Shahriari et al., 2019).

Specifically, Viol release from the NLC during nanoparticle disintegration can be affected because the hydrophobic character of the biodye could be stuck to the lipids, and consequently Viol bioavailability will be reduced. In this sense, the inclusion of an inactive but dormant “alive” lipase in the NLC can improve Viol release from the lipids by hydrolyzing the ester linkages of the lipids in the presence of a physiological environment. Lipase from *R. miehei* showed optimum activity at temperatures between 37°C and 40°C, in the presence of surfactants, but the optimal pH is strain-dependent and goes from the alkaline range (i.e., pH between 8.0 and 8.8) to the acid one (i.e., pH = 5.0–5.4) (Takó et al., 2017). Both simultaneous conditions are relevant for cancer treatment since solid tumors show high temperatures compared to the nearby normal tissue because of high metabolic rates, and also an acid surrounding environment due to the exacerbated production of lactic and acetic acids (Rahim et al., 2021). In addition, lipase released from the NLC can act on the membrane surface of cancer cells, not only disturbing its structure and function but also hydrolyzing lipids that could facilitate the diffusion of Viol into the cells. Moreover, the presence of lipase released from the NLC could catalyze the hydrolysis of triglycerides present in the serum, reducing the formation of precancerous lesions as well as colon, pancreatic, and prostate cancers (Chandra et al., 2020).

On the other hand, medical implants provide a novel platform from which the drug can be released over time. Medical implants show at least two obvious advantages: first, the release is site-specific (or locoregional), reducing the adverse side effects resulting from the systemic drug circulation and allowing a better quality of life for the patient since no other tissues and organs will be in contact with the drug. Second, they guarantee the patient’s adherence to the treatment (Ahmed et al., 2019). In the specific case of solid tumors, whenever possible, resection surgery is the first treatment option, but combined therapies are key in cancer treatment, and surgery is frequently associated with adjuvant chemotherapy treatments (Wolinsky et al., 2012).

Three-dimensional bioprinters can be used for the development of organoids, tissue engineering, scaffolds for drug delivery, tissue printing, and novel hybrid devices (Stanisz et al., 2020). Particularly, a 3D bioprinter was designed and constructed in our laboratory to develop different matrix structures for drug delivery used as patches or implants for the treatment of different pathologies (Katz and Castro, 2019). The treatment of solid tumors with 3D-bioprinted matrices has the advantages of personalized medicine because it is possible to adjust the implant to the characteristics, size, and location of the tumor. In addition, 3D implants can be placed as close as possible to the tumor surfaces adjusted to the tumor microenvironment or also in the cavity left by the resected tumor after a surgery that reduces the drug circulation in the body and consequently, the administered drug amount and its adverse effects.

Two biopolymers were selected for the development of a 3D-bioprinted matrix: chitosan (Chi) and hydroxypropyl methylcellulose (HPMC) because of their advantages. Chi is composed of ß-linked N-acetyl-D-glucosamine and D-glucosamine randomly distributed. Chi possesses a residual positive charge making the biopolymer mucoadhesive and cell-adhesive, very relevant properties to enhance the attachment of the biopolymer gels to the cell surfaces, which increases tissue permeability and drug residence time. In addition, Chi displays antimicrobial and antioxidant activities. Chi is nontoxic, biocompatible, and biodegradable by mammalian enzymes, making the biopolymer an excellent candidate for biomedical applications in the form of gels, nanoparticles, fibers, and films (Shariatinia and Jalali, 2018).

Cellulose is the most abundant biopolymer found in nature and is composed of linear ß-D-glucose units of different lengths, but its limited solubility reduces applications. HPMC is a derivate of methylcellulose containing propylene glycol ether. Also, HPMC is a nontoxic polymer, considered generally recognized as safe (GRAS) by the FDA, and approved as a food additive by the EU (Siepmann and Peppas, 2012).

In the present work, a platform of stimuli-responsive controlled release system (SRCRS) based on NLC containing lipase as a trigger agent and covered with Chi was integrated into a polymeric matrix composed of Chi and HMPC. The composite polymeric matrix was optimized for stability and to be extruded by a 3D bioprinter developed and constructed in our laboratory. The purpose of the 3D-printed matrix was to be used as an implant. Violacein was selected as a hydrophobic drug model entrapped in NLC for the potential treatment of solid tumors. The matrix system was characterized using biophysical techniques, TEM, FTIR, TGA, and dispersive light. The release of Viol from the matrices was studied by structured kinetic models. Cytotoxicity *in vitro* was studied in A549 and HCT116 cancer cell lines.

## 2 Materials and Methods

### 2.1 Materials

Violacein [3-(1,2-dihydro-5-(5-hydroxy-1*H*-indol-3-yl)-2-oxo-3*H*-pyrrol-3-ylidene)-1,3-dihydro-2*H*-indole-2-one] was synthesized by *Chromobacterium violaceum* CCT 3496 and used following earlier techniques reported elsewhere. Briefly, an isolated colony of *C. violaceum* from an agar plate was inoculated in 250 ml Erlenmeyer containing 100 ml of a medium composed of 5 g l^−1^ D-glucose, 5 g l^−1^ peptone, and 2 g l^−1^ yeast extract dissolved in MiliQ water. The vessel was incubated at 150 rpm on a rotatory at 30°C for about 36 h. Later the bacterial culture was centrifuged at 10,000 × g for 15 min at 5°C, and the supernatant was discarded. Violacein was purified from the cells using a Soxhlet extractor. The Viol purity was 98% purity determined spectroscopically (data not shown) (Mendes et al., 2001; Berti, et_al., 2019).

Lipase, from *R. miehei* lyophilized powder (Lip), 4-nitrophenyl palmitate (pNPP), Poloxamer 188 (Kolliphor® P188), chitosan (Chi, poly (D-glucosamine) deacetylated chitin, MW ≈ 161 kDa), and (hydroxypropyl)methylcellulose (HPMC, MW ≈ 26 kDa) were bought from Sigma-Aldrich (Buenos Aires, Argentina). Myristyl myristate a solid lipid at RT (MM, Crodamol® MM, MP = 36–40°C), and cetyl palmitate an oily triglyceride with saturated fatty acids (Crodamol® GTCC-LQ, MP = −4°C) were a generous donation of CRODA® (Argentina). All other chemicals, solvents, and reagents employed in the present work were of analytical grade.

### 2.2 Lipase Activity and Stability Measurements

Lipase activity was measured by the release of the colored compound 4-nitrophenol from 4-nitrophenyl palmitate (pNPP) as previously reported (Romero et al., 2012). Briefly, pNPP was pre-dissolved in 1:4 acetonitrile-isopropanol supplemented with 0.1% (w/v) Triton X-100. The pNPP solution was dispersed in a 10 mM buffer supplemented with 0.2% (w/v) Arabic gum to reach a final concentration of 500 µM. One ml of pNPP was placed in a 48-well plate to run the enzymatic reaction. A lipase sample was added, and the solution’s absorbance was measured at 405 nm at 37°C every minute for 20 min to determine 4-nitrophenol concentrations. Lipase activity was displayed as µmol of 4-nitrophenol released per second.

The stability of lipase was determined at different pH values by incubating the enzyme in different buffers at room temperature, and then its activity was evaluated using buffer phosphate (pH = 7.4) and 37°C for 1 h.

### 2.3 Nanostructured Lipid Carrier and Nanostructured Lipid Carrier With Lipase Preparation

NLCs were synthesized by ultrasonication (Rodenak-Kladniew et al., 2017). Concisely, a lipid phase containing 400 mg of the solid lipid myristyl myristate (MM), 100 µl of a commercial mixture of caprylic and capric acids (Crodamol® GTCC), and 20 µmol Viol was melted in a 70°C bath. Later, 20 ml of 4.0% (w/v) poloxamer 188 adjusted to pH = 5.0 with acetic acid/sodium acetate buffer was mixed with the lipid phase and sonicated in an ultrasonic processor (130 W, Cole-Parmer, United States) at 70% amplitude for 15 min.

The Viol remaining in the NLC was assayed in a 500 µl sample of the NLC-Viol formulations taken and filtered in 100 kDa cut-off filters (Amicon® Ultra- 0.5 ml, Merck Millipore, Ireland). The absorbance of the filtered solution was measured at 580 nm.

For formulations containing lipase (Lip), 5.0 ml of NLC dispersion was incubated with enough lipase to produce an activity of 2.0 µmol of 4-nitrophenol/s at 5°C overnight with an agitation of 150 rpm with a magnetic stirrer.

### 2.4 Nanoparticle Measurements

The average hydrodynamic diameter (D_H_), polydispersity index (PdI), and Z-potential (*ζ*) were determined for NLC-Viol and NLC-Viol-Lip samples diluted 1/100 [2.0% (w/v) initial concentration and 0.02% (w/v) final concentration of MM] in ultrapure water using a Zetasizer Nano ZS series (Malvern Instruments, United Kingdom). The determinations were performed at a 633 nm (He-Ne) laser at a 173° measurement angle at 25°C by triplicate.

Transmission electron microscopy (TEM) images of the nanoparticles were acquired in a Jeol-1200 EX II-TEM microscope (Jeol, Columbia, MD, United States). NLC suspensions were diluted 1/100 [2% (w/v) and 0.02% (w/v) initial and the final concentration of MM, respectively] in ultrapure water or 10 mM phosphate buffer (pH = 7.4) followed by spreading a 10 µl sample on a 400 mesh Cu grid. The excess sample was removed with filter paper. A phosphotungstic acid drop was added to the samples for contrast enhancement.

### 2.5 Biopolymer Ink Preparation and Extrusion

A weighted amount of 200 mg chitosan was hydrated in 1.0 ml of 10% acetic acid to form a homogenous paste. Later, 1.5 ml of NLC formulation with or without Lip was added and mixed, and then, 500 mg of HPMC was added, and the ink was thoroughly mixed and charged in a sterilized 5 ml syringe with Luer lock (TERUMO®, Philippines). The loaded syringe was later centrifuged at 7,000 × g for 20 min to eliminate any air bubbles from the 3D ink. A conic plastic tip or nozzle (Fish Dispensing, China) with a 0.41 mm exit orifice was attached to the syringe before extrusion.

The 3D meshes were produced by extrusion in a 3D printer designed in our laboratory. Briefly, the 3D printer consists of an extruder that precisely pushes ink out of the syringe, a positioning system for the movements on three axes, and a controller. The extruder is composed of a socket for the syringe and a bipolar Nema 17 stepper motor with a high precision planetary system; the movement on the three axes is provided by two bipolar Nema 17 stepper motors coupled with 8 mm THSL screws and anti-backlash nuts. The open-source electronics used contain a 16 MHz ATmega 2560 processor that is commanded with Marlin firmware.

**FIGURE 1:**
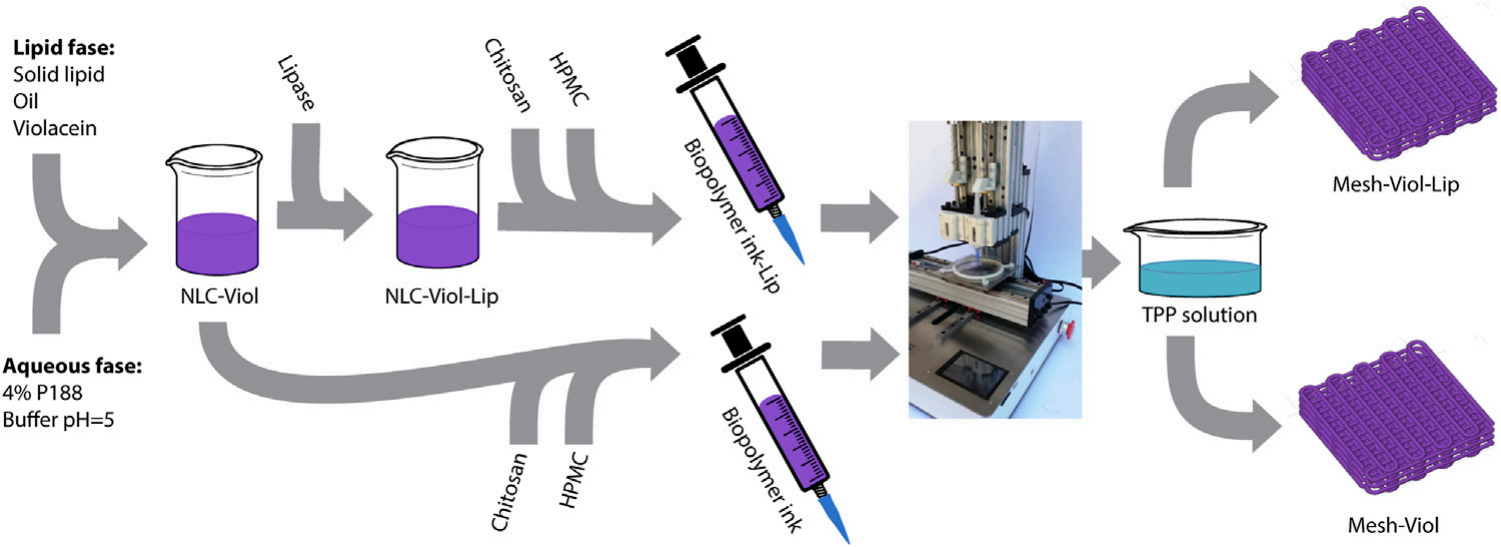
Simplified workflow to produce the 3D meshes.

The 3D meshes produced by 3D bioprinting were submerged for 10 min in a 3.0% tripolyphosphate (TPP) solution, washed with ultrapure water, and saved in the fridge for biophysical characterization. Samples of TPP solution and ultrapure water used for washing were taken to determine Viol concentration. Figure 1 shows a simplified workflow of NLC and 3D-mesh production.

### 2.6 Entrapment Efficiency of the Meshes

Entrapment efficiency was measured for both matrices prepared with NLC-Viol (Mesh-Viol) and meshes prepared with NLC-Viol-Lip (Mesh-Viol-Lip). This was conducted by two methods. The indirect method was carried out by measuring Viol in the TPP solution and in the water used to wash the 3D meshes, and then subtracting the violacein amount in those solutions from the initial amount of drug, using the following equation: Eq. 1

$$EE_{indirect}\left(\%\right)=\frac{mViol_{i}-\left(cViol_{TPPsol}\times V_{TPPsol}\right)-\left(cViol_{ww}\times V_{ww}\right)}{mViol_{i}}\times 100\%$$ (1)

where *mViol*_*i*_ is the initial amount of Viol present in the preparation, *cViol*_*TPPsol*_ is the concentration of violacein in the TPP solution, *cViol*_*ww*_ is the concentration of Viol in the ultrapure water after washing, and *V*_*TTPsol*_ and *V*_*ww*_ are the volumes of those solutions.

On the other hand, the direct method consisted in adding 3.0 ml of ethanol to a weighted amount of Mesh-Viol or Mesh-Viol-Lip followed by sonication in an ultrasonic cleaning bath for 30 min. The procedure was performed twice with ethanol replacement. The efficiency was then calculated by the following equation: Eq. 2

$$EE_{direct}\left(\%\right)=\frac{cViol_{1}\times V_{1}+cViol_{2}\times V_{2}}{mViol_{i}}\times 100\%$$ (2)

where *mViol*_*i*_ is the initial amount of Viol added to the preparation, *cViol*_*1,*_ and *cViol*_*2*_ are the concentration of Viol in the first and second ethanol extraction, and *V*_*1*_ and *V*_*2*_ are the volumes of those solutions.

### 2.7 Violacein Release From the 3D-Printed Meshes

Four meshes were weighed and placed in 30 ml of 10 mM phosphate buffer at pH = 7.4 and 37°C, in duplicate. Every hour a 1.0 ml sample of the media was taken and replaced with an equal volume of fresh buffer. The samples were later filtered in 100 kDa cut-off filters (Amicon^®^ Ultra- 0.5 ml, Merck Millipore, Ireland). Filtered solution absorbance was determined at 580 nm to evaluate Viol’s release. The results were expressed as a fraction of a total load of Viol released versus time. The Viol release profiles were analyzed by structured kinetic release models (Supplementary Table S1).

### 2.8 Physicochemical Characterization of the 3D Meshes

#### 2.8.1 Fourier Transform Infrared Spectroscopy

FTIR spectra were performed in freeze-dried in a Nicolet 6700 model (Thermo Scientific CT, United States) spectrometer coupled with an attenuated total reflectance (ATR) accessory. Scans were performed 32 times for each sample in the range from 600 to 4,000 cm^−1^ with a 4 cm^−1^ resolution.

#### 2.8.2 X-Ray Diffraction

Diffraction patterns of the freeze-dried samples were obtained in an Analytical Expert instrument using Cu-K radiation (*θ*= 1.54 Å) from 2θ = 10° to 70° in continuous mode with 0.07° step size.

#### 2.8.3 Thermal Analysis

Thermogravimetric analysis (TGA) was performed in a Shimadzu TGA-50 instrument. All assays were carried out in the range of 20°C–900°C at a heating rate of 10°C/min under an N_2_ atmosphere.

Differential scanning calorimetry (DSC) was carried out on a Perkin Elmer Inc., Model Pyris 1 (Waltham, MA, United States) instrument under an N_2_ atmosphere. The heating rate of 10°C/min was used in the range of 0–250°C. All samples were previously frozen-dried.

### 2.9 Scanning Electron Microscope Image Acquisition

SEM analysis of 3D-printed meshes was run with freeze-dried samples that were then covered with a gold layer thickness of 15–20 nm using a Balzers SCD 030 metallizer. Images were obtained in a Philips SEM 505 scanning electron microscope and processed in an image digitizer program (Soft Imaging System ADDA II, SIS). Later, the images were analyzed with ImageJ^®^ software (NIH, United States), and the surface roughness was estimated by the standard deviation of grey values in the image histogram (Wang et al., 2005).

### 2.10 Cytotoxic Studies

Human colorectal carcinoma HCT-116 and lung adenocarcinoma epithelial A549 cells were cultured in Minimal Essential Medium (MEM, HyClone, CA, United States) supplemented with 10% fetal bovine serum (*Internegocios* S.A., Argentina) and 1.0% penicillin/streptomycin (Gibco Invitrogen Corporation, United States) in 5.0% CO_2_ at 37°C. The viability of the cells was assayed using MTT [3-(4,5-dimethylthiazol-2-yl)-2,5-diphenyltetrazolium bromide] assay (Mosmann, 1983). HCT-116 and A549 cells (4.0 × 10^3^ cells/ml) were seeded in 96-well plates and incubated under standard conditions for 24 h. Then, the cells were exposed to 0.5, 2, or 4.0 µM of free Viol (prepared in DMSO lower than 0.05% (v/v) final concentration), or equivalent amounts (µM of Viol or mg/ml of MM) of NLC and NLC-Lip with and without Viol for 24 h. The medium was discarded and incubated in the presence of 100 µl of 500 μg/ml MTT solution prepared in MEM with 3 h incubation. Formazan was dissolved in 100 µl DMSO. The microplates were stirred for 5 min and readings of absorbance at *λ* = 560 nm were determined in a microplate reader (Beckman Coulter DTX 880 Multimode Detector, United States). A reduction in cell viability by more than 30% was considered a cytotoxic effect (Doktorovova et al., 2014). A conditioned medium was used to assay Mesh-Viol and Mesh-Viol-Lip. That is, meshes with and without viol, and with and without Lip were incubated in MEM supplemented as before for 48 h. Then, the media was used to incubate A549 and HCT116 cells for 24 h. Later, MTT colorimetric assays were performed as previously described.

## 3 Results and Discussion

### 3.1 Lipase Activity and Nanostructured Lipid Carrier Preparation

**FIGURE 2:**
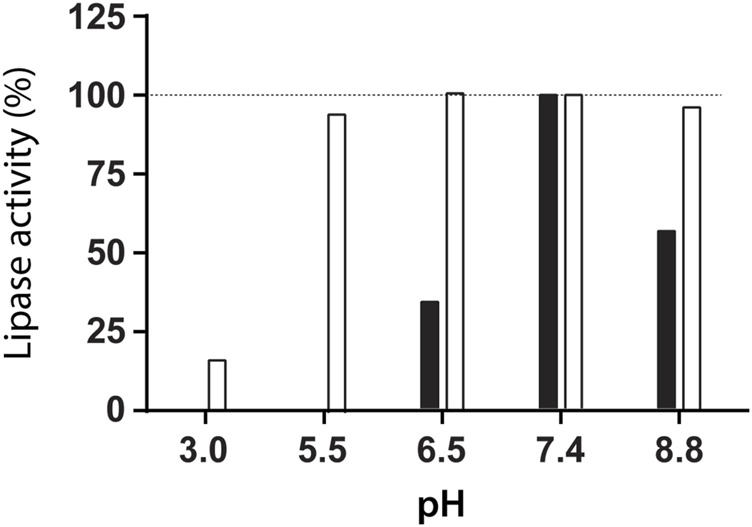
Lipase activity measured at pH = 7.4 and 37°C after incubation for 6 h at room temperature at the indicated pH (white, □) and lipase activity assayed at the indicated pH and 37°C (black, ■).

The lipase activity was evaluated at different pH values at room temperature. The high activity of lipase from *R. miehei* was detected at pH = 7.4, but no enzyme activity was observed at pH = 5.5 (Figure 2). However, 95% of enzyme activity was recovered at pH = 7.4 after lipase incubation at pH = 5.5 for 6 h, indicating a reversible enzyme inhibition. Based on this result, the synthesis of NLC containing lipase was performed at pH = 5.5.

Encapsulation efficiency of Viol in the NLC-Viol without and with lipase was near 100% since Viol concentration in the filtered solution was below the limit of detection (Supplementary Material Table S3).

### 3.2 Nanostructured Lipid Carrier Size and Z-Pot

The average hydrodynamic diameter (D_H_), PdI, and Z-Pot (*ζ*) are shown in Supplementary Table S2. The addition of lipase does not significantly alter the mean particle size (154.3 nm for NLC-Viol and 151.5 nm for NLC-Viol-Lip; *p* > 0.05), but it does significantly alter the PdI (0.241 for NLC-Viol and 0.319 for NLC-Viol-Lip; *p* < 0.05). The *ζ* value is close to zero, as might be expected from the fact that the surfactant used to stabilize NLC, P188, is neutral in charge. The *ζ* change between formulations with and without lipase is significant (*p* < 0.05) but still very small. Results are summarized in Supplementary Table S2.

**FIGURE 3:**
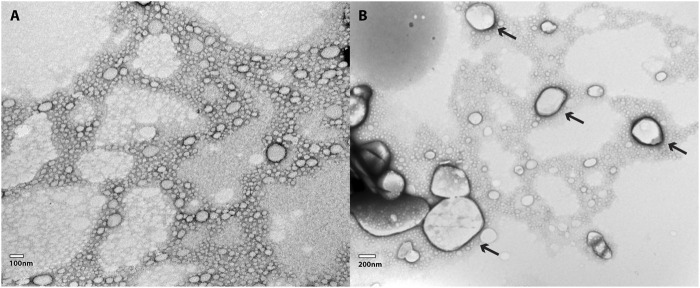
**(A)** TEM micrography of a 1:100 dilution of NLC-Viol-Lip, and **(B)** TEM micrography of a 1:100 dilution of NLC-Viol-Lip after it was incubated at a pH = 7.4 for 6 h. Arrows in **(B)** point aggregates.

TEM images for NLC-Viol-Lip before and after incubation in a 10 mM phosphate buffer (pH = 7.4) for 6 h are shown in Figure 3. Before incubation NLC-Viol-Lip showed an average diameter of 93.6 nm based on 36 measurements from micrographs (Figure 3A). After incubation aggregates were observed (Figure 3B, arrows). These aggregates can be explained since lipase activity probably weakens the structure of the nanoparticle by catalyzing lipid hydrolysis, and therefore remnant NLC produces aggregate structures.

### 3.3 Biopolymer Ink Preparation and Extrusion

Different proportions of Chi and HPMC were tested to create an appropriate polymer mixture to be extruded by the 3D bioprinter. The final polymer concentrations for the bioink were 6.6% (w/w) Chi and 16.6% (w/w) HPMC, equivalent to HPMC/Chi: 2.5 ratio. Lower HPMC content resulted in an unstable printed bioink, while higher HPMC concentrations drastically reduced the cross-linking with TPP.

Maximum printing speed that did not produced errors in ink continuity found was 400 mm/min. For the nozzle diameter used (0.41 mm) a layer height of 0.33 mm was found optimal, since layer heights less than that, caused the nozzle to damage the lower layer and larger layer sizes prevented the correct adhesion of the developing layer on the lower layer. A video illustrating the printing process can be found in Supplementary Material.

The two methods used to estimate EE (%) gave differences lower than 10% among them (Supplementary Table S3). However, the average EE (%) of Mesh-Viol and Mesh-Viol-Lip was 93.8% and 92.3%, respectively. These values are very close considering the several experimental steps for the production of the drug delivery devices (i.e., sonication, mixing, cross-linking, and washing).

### 3.4 Violacein Release From the Meshes

**FIGURE 4:**
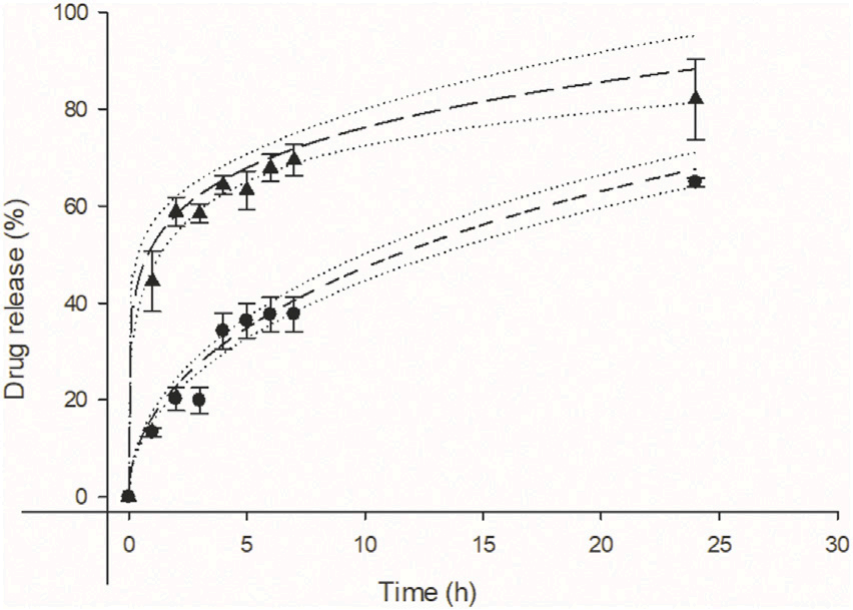
Violacein kinetic release from mesh (●), and mesh containing lipase (▴) at pH = 7.4 respectively. Baker and Lonsdale model fit for mesh (**− −,** R^2^ = 0.95) and Korsmeyer-Peppas model fit (**−− −−,** R^2^ = 0.95) for mesh containing lipase. Upper and lower 95% confidence interval for each fit (·····).

The difference between the two Viol release profiles of matrices with or without lipase is obvious (Figure 4). Lipase strongly stimulates the release of Viol from the polymeric matrix, reaching 60% release at pH 7.4 after 2 h incubation. While only 20% of Viol was released from the matrix without lipase under the same experimental conditions. The kinetic release of Viol from Mesh-Viol and Mesh-Viol-Lip was analyzed with typical structured kinetic models (Supplementary Table S1). The best correlations of Viol release were found with Higuchi (R^2^ = 0.94), Korsmeyer-Peppas (R^2^ = 0.95) and Baker-Lonsdale (R^2^ = 0.95) tested models for the matrix without lipase. While the best correlation of Viol release from the matrix containing lipase was observed in the Korsmeyer-Peppas model (R^2^ = 0.95) (Supplementary Table S5). The kinetic of Viol release from the matrices and Baker-Lonsdale fit for Mesh-viol and Korsmeyer-Peppas for Mesh-Viol-Lip are displayed in Figure 4. The “n” parameter in the Korsmeyer-Peppas model can be used to predict the potential mechanism involved in the drug release (Supplementary Table S1). In this case, Mesh-Viol fitted an *n* = 0.17 and Mesh-Viol-Lip an *n* = 0.46; both cases fall below *n* < 0.5 condition. Considering these experimental values, the Korsmeyer-Peppas model predicts a Viol release based mostly on Fick’s law, i.e., only based on the diffusional mechanism (Ritger and Peppas, 1987).

Additionally, Viol releases from the Mesh-Viol and Mesh-Viol-Lip matrices were performed at pH = 5.0. No significant differences were found between the Viol release in both matrices (Supplementary Figure S1). These results are indicative of the relevant lipase role in Viol release. However, the Viol release was faster than in pH = 7.4 buffer, with around 65% of the drug released in the first 2 h, which can be expected since Chi solubility is greatly enhanced at lower pH (Kou et al., 2017).

### 3.5 Mesh Physicochemical Characterization

#### 3.5.1 Scanning Electron Microscope Micrograph Results

**FIGURE 5:**
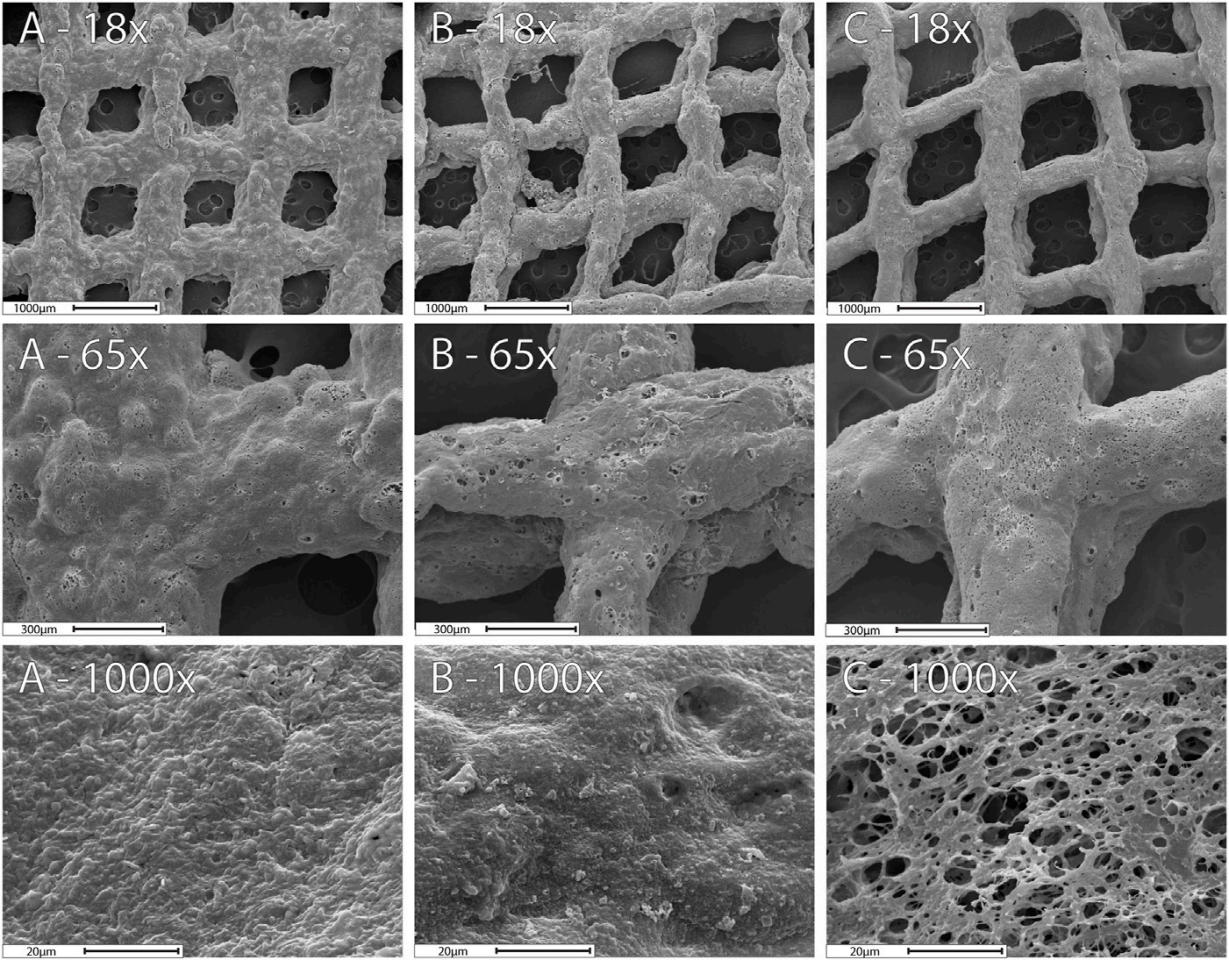
SEM images for the 3D-Mesh previous release **(A)**, Mesh-Viol and Mesh-Viol-Lip released in phosphate buffer (pH = 7.4) after 24 h incubation **(B)** and **(C)** respectively.

The SEM images display the matrix structure after exposing the 3D prints to 10 mM phosphate buffer (pH = 7.4) for 24 h (Figure 5). The SEM images showed a relaxed matrix structure with mesh gaps expanded, determined by the ImageJ software (Supplementary Table S4). Meshes before drug release did not exhibit any significant difference (*p* > 0.05) and are presented as one single group. However, changes in the structure of the meshes with and without Lip after Viol release were significant (*p* < 0.05). The Mesh-Viol-Lip displayed a big variation in the matrix structure that can be attributed to the presence of lipase in the matrix. This loosening in polymeric structure can also be observed in the microstructure at higher magnifications (×1,000) (Figure 5). Mesh-Viol-Lip shows a larger number of cavities between polymer areas. Also, the roughness of Mesh-Viol-Lip estimated by the grey histogram confirmed this result (Supplementary Table S4). A comparative analysis of the standard deviations of the matrices showed that Mesh-Viol and Mesh-Viol-Lip before drug release possess smother surfaces (SD = 35.5) than Mesh-Viol or Mesh-Viol-Lip after the release (SD = 40.1 and 48.3 respectively).

#### 3.5.2 X-Ray Diffraction Analysis

**FIGURE 6:**
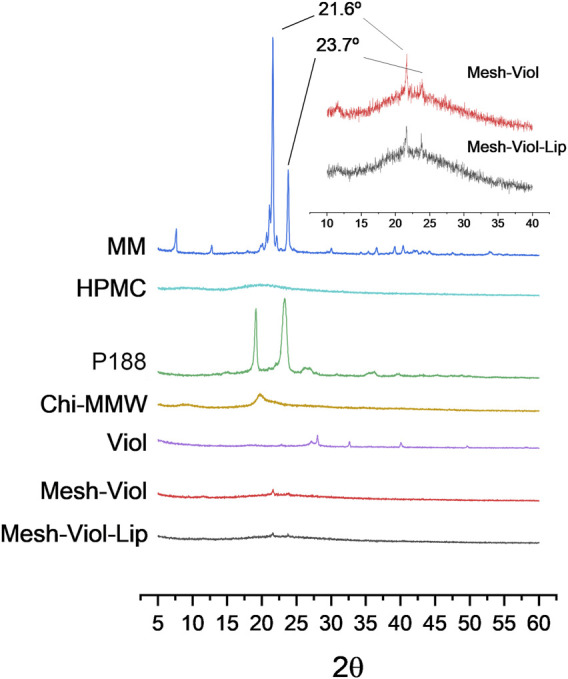
Diffractograms for Mesh-Viol-Lip, Mesh-Viol, MM (myristyl myristate), Chi-MMW (medium molecular weight chitosan), HPMC (hydroxypropyl methylcellulose), and P188 (Poloxamer P188). Mesh-Viol and Mesh-Viol-Lip were incubated for Viol release in phosphate buffer (pH = 7.4) for 24 h before performing the analysis. Inset is a detail for Mesh-Viol-Lip and Mesh-Viol between 10° and 40°.

Figure 6 shows XRD patterns for both 3D meshes after 24 h incubation in release buffer (pH = 7.4) as well as their pure components. P188 showed a high degree of crystallinity when pure, with two intense and characteristic peaks at 19.1° and 23.3°, and a dimmer broader peak between 25.5° and 27.0° (He et al., 2011). Chi showed a small peak at 19.7° and a dim broad peak at 9°, while HPMC exhibited a wide peak between 15° and 25°. However, none of these peaks appear in the mixtures, indicating a loss of the crystalline structure of the polymers in the composite. Mesh-Viol and Mesh-Viol-Lip show a small compound peak at 21.6° and 23.7° (Figure 6 inset); these peaks also appear and are characteristic of the MM diffraction pattern, although in much higher intensity, which indicates that a few amounts of MM remain in crystals after NLC preparation.

#### 3.5.3 Fourier Transform Infrared Analysis

**FIGURE 7:**
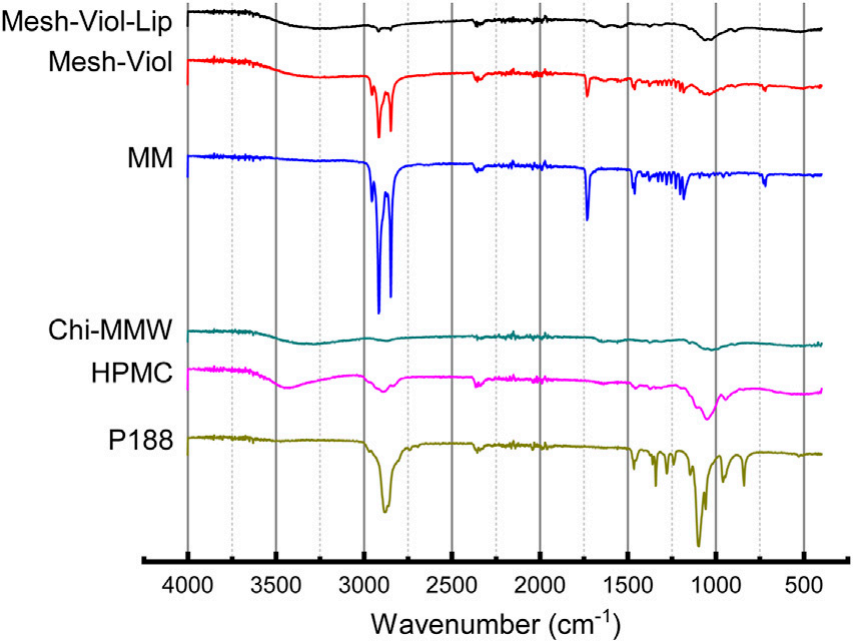
FTIR spectra for Mesh-Viol-Lip, Mesh-Viol, MM (myristyl myristate), Chi-MMW (medium molecular weight chitosan), HPMC (hydroxypropyl methylcellulose), and P188 (Poloxamer P188). Mesh-Viol and Mesh-Viol-Lip were incubated in release buffer (pH = 7.4) for 24 h before performing the analysis.

FTIR spectra for both 3D meshes as well as their major constituents incubated in phosphate buffer (pH = 7.4) for 24 h are displayed in Figure 7. P188 showed an intense peak at 2,877.32 cm^−1^ attributed to C-H aliphatic stretching, a smaller peak at 1,342.23 cm^−1^ attributed to in-plane O-H bending, and a sharp peak at 1,099.24 cm^−1^, and a smaller peak at 960.39 cm^−1^ corresponding to ether C-O symmetric and asymmetric stretching vibrations (Lal and Datta, 2014). HPMC spectrum shows a broad peak between 3,100 and 3,600 cm^−1^ corresponding to O-H stretching vibrations, 2,894.67, and 1,049.10 cm^−1^ in agreement with C-H and C-O stretching respectively (Furqan et al., 2017). Chi also showed a broad O-H stretching vibration band between 3,100 and 3,600 cm^−1^, but partially overlapped with broadband due to asymmetric/symmetric N-H bonds. The band at 2,871.53 cm^−1^ was assigned to C-H axial stretching, while the 1,641 cm^−1^ band was attributed to -C=O stretching in the acetamide groups, and the 1,556 cm^−1^ to the trans-secondary amides (Acosta-Ferreira et al., 2020; Peng et al., 2020); additionally, the 1,019 cm^−1^ band was attributed to C-N primary amine stretch (Peng et al., 2020). MM showed three sharp peaks at 2,848.39, 2,915.88, and 2,954.45 cm^−1^, attributed to asymmetric, symmetric stretching of C-H bonds on −CH_2_ and C-H bond asymmetric stretching on −CH_3_ groups respectively. Another relevant sharp peak was found at 1731.8 cm^−1^, corresponding to ester carbonyl stretching vibrations (Castro et al., 2021). Finally, both 3D meshes showed a complex broad peak between 1,150 and 900 cm^−1^, from overlapping P188, HPMC, and Chi peaks in that band of the spectrum, and broadband between 3,000 and 3,500 cm^−1^ from O-H and N-H vibrations in HPMC and Chi. However, the 3D meshes containing Lip (Mesh-Viol-Lip) exhibited weaker peaks at 2,848.39 and 2,915.88 cm^−1^, the bands presented by MM. Furthermore, Mesh-Viol-Lip did not show the characteristic peak at 1731.8 cm^−1^ attributed to −C = O stretching of the ester group, while it appeared in the 3D mesh without Lip (Mesh-Viol). All these points could reflect a loss in MM content in Mesh-Viol-Lip due to lipase activity in the 3D mesh.

#### 3.5.4 Thermogravimetric Analysis

**FIGURE 8:**
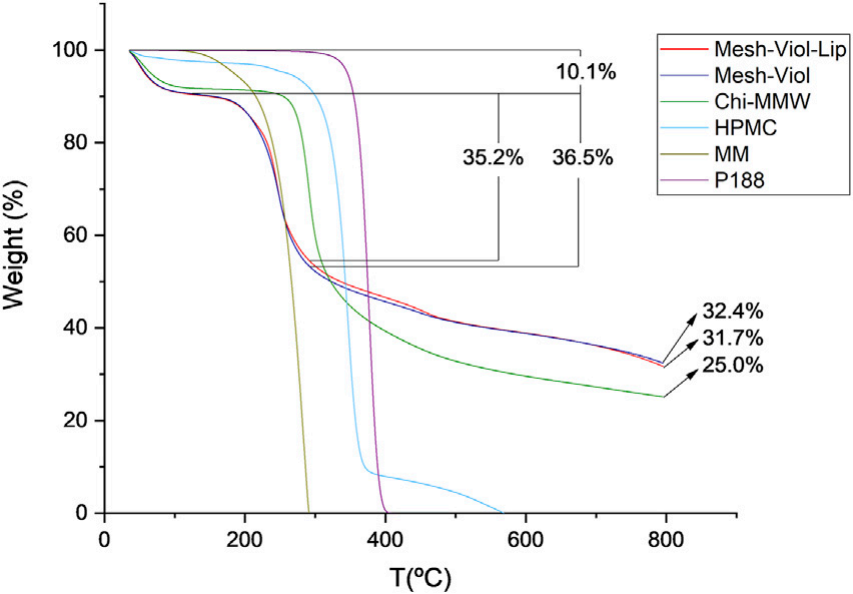
Thermogravimetric analysis for Mesh-Viol-Lip, Mesh-Viol, MM (myristyl myristate), Chi-MMW (medium molecular weight chitosan), HPMC (hydroxypropyl methylcellulose), and P188 (Poloxamer P188). Mesh-Viol and Mesh-Viol-Lip were incubated in phosphate buffer (pH = 7.4) for 24 h before performing the analysis.

The thermal stability of the 3D meshes after release was studied. Figure 8 shows the percentage mass loss versus temperature. Both 3D meshes lose 10% of their weight between 0°C and 125°C, which is due to remnant water in the samples; similarly, Chi and HPMC lose 9% and 3% of their weight respectively.

The maximal HMPC weight loss was at 346°C, and before 400°C more than 90% of its mass was lost. This process is normally attributed to the decomposition of cellulose esters, and volatilization of these degraded residues (Hasanin et al., 2022). On the other hand, Chi-MMW, weight loss after 250°C is due to further dehydration, deacetylation, and depolymerization (de Britto and Campana-Filho, 2007). MM maximum weight loss was found at 283°C; this step is normally attributed to volatilization rather than thermal degradation (Xu et al., 2022).

Between 125°C and 290°C Mesh-Viol and Mesh-Viol-Lip lose 36.5% and 35.2% of their mass respectively, which coincides with the acute mass loss of MM. Moreover, after finishing the essay, the Mesh-Viol has a remnant of 32.4% of its weight, 31.7% of Mesh-Viol-Lip, and 25.0% of Chi. Since none of the other constituents remain at this temperature, the meshes exhibit greater thermal stability than pure materials, suggesting a chemical interaction between the biopolymers. In the Supplementary Material, a figure showing weight derivative (%) versus temperature is provided (Supplementary Figure S2).

#### 3.5.5 Differential Scanning Calorimetry Analysis

**FIGURE 9:**
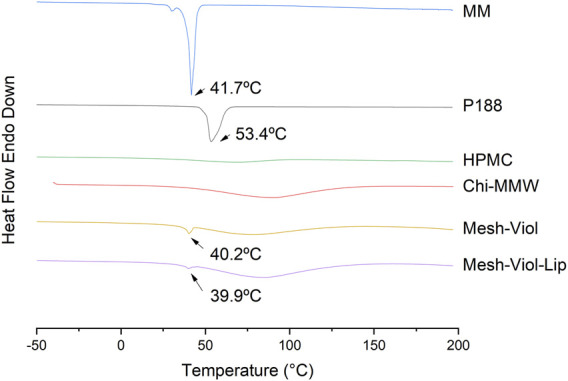
DSC thermograms for Mesh-Viol-Lip, Mesh-Viol, MM (myristyl myristate), Chi-MMW (medium molecular weight chitosan), HPMC (hydroxypropyl methylcellulose), and P188 (Poloxamer P188). Mesh-Viol and Mesh-Viol-Lip were incubated in phosphate buffer (pH = 7.4) for 24 h before performing the analysis. Y-axis represents heat flow (exothermic is up).

The heat flow from the 3D mesh samples and their main constituents versus temperature, “up” curves in the Y-axis representing the exothermic process, and “down” curves showing the endothermic process, are displayed in Figure 9. MM shows a sharp intense peak at 41.7°C, corresponding to its melting temperature (T_m_), with an area under peak, that is, a melting enthalpy (ΔH_m_) of -205 J/g, both results are consistent with the bibliography (Aydn and Okutan, 2011), a small shoulder can be attributed to a pleiomorphism transition. Similarly, T_m_ for P188 is 53.4°C (He et al., 2011), and a ΔH_m_ of −145 J/g. The broad endothermic peak between 50°C and 100°C in HPMC and Chi-MMW can be attributed to a loss of water (El Maghraby and Alomrani, 2009). DSC analysis for mixtures, Mesh-Viol and Mesh-Viol-Lip showed a sharp and small peak near MM T_m_, at 40.2°C and 39.9°C respectively. This T_m_ depression can be explained by the Gibbs-Thomson equation (Supplementary Equation S1) and (Supplementary Material) (Han et al., 2008). Based on the Gibbs-Thomson equation, as the radius of the nanoparticle decreases, so does its melting point. Also, a bigger and wider peak appears before 100°C. Mesh-Viol-Lip small peak was smaller, while the big broad peak was bigger, which may indicate a change in Mesh lipid and water content after drug release.

### 3.6 Cytotoxicity Assays

**FIGURE 10:**
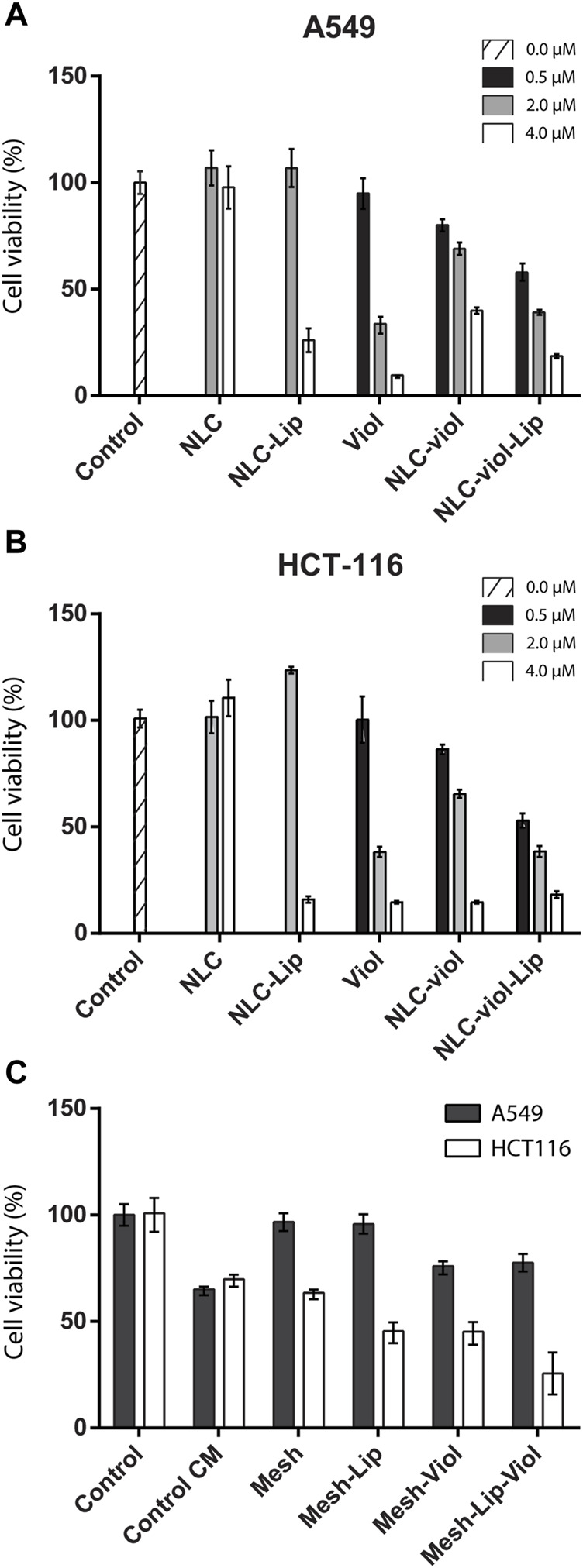
Cell viability For NLC formulations and free Viol in A549 **(A)** and HCT-116 **(B)** cell lines, respectively. Cell viability on conditioned medium **(C)**.

Figure 10 A and B show cytotoxicity for different NLC formulations and free Viol. At 0.5 µM (filled in black) NLC-Viol showed a greater effect than free Viol. On the contrary, the cytotoxic effect was greater in 2.0 µM free Viol (filled in grey). This was observed for both cell lines tested. This phenomenon was already reported in previous works where Viol was nanovehiculized (Berti, et_al., 2019; Berti et al., 2022).

Additionally, in both cell lines tested NLC-Viol-Lip showed more cytotoxicity at 2.0 µM (filled in grey) than NLC-Viol (*p* < 0.05), even though at this concentration lipase and empty NLC showed no toxic effects. This result revealed that a more efficient biocide effect between NLC, Lip, and Viol could be attributed to the increase of free biodye due to nanoparticle degradation by lipase activity. However, other effects could contribute to this result. Recently, Viol was described as a membrane disruptor in an attempt to explain its various biological effects (Gupta et al., 2021). Considering this hypothesis, the activity of lipase and the activity of violacein could produce a positive effect at their site of action. Additionally, the incorporation of Lip into the formulation can impact positively on the treatment of solid tumors since it was found that lipase activity is suppressed or absent in some breast, colorectal, and lung tumors; therefore, lipase activity may have a tumor-suppressive effect (Sun et al., 2013).

Figure 10C shows the cytotoxicity assay for A549 and HCT116 cell lines when they were incubated in a medium that was in contact with 3D-printed meshes. CM-control represents a medium without treatment, that is, without 3D-printed mesh, which was incubated for the same period as the treated media. This experimental procedure was considered the “aging” of the medium alone, which presents a reduction in viability in the HCT116 cell line (*p* < 0.005). However, the biopolymeric matrix alone without violacein or lipase (Mesh) did not further reduce viability when compared with CM-control (*p* > 0.5). Besides, both the lipase effect (Mesh-Lip) and violacein effect (Mesh-Viol) significantly decreased the cell viability (*p* < 0.005). Additionally, the major cytotoxic effect on the HCT116 cell line was observed with Mesh-Lip-Viol, being greater than that of Mesh-Lip or Mesh-Viol (*p* < 0.05 in both cases).

## 4 Conclusion

Recent strategies for the treatment of several diseases could involve the use of enzymes, with more than 100 potential candidates. However, marketed enzymes applied in biomedicine are still scarce (Bosio et al., 2016). The emergence of nanotechnology opened a new landscape for the development of efficient devices used in the treatment and diagnosis of diverse pathologies, particularly cancer. In this regard, diverse approaches of nanoparticles containing enzymes to target tumors were recently reviewed (Li et al., 2020; Kapalatiya et al., 2022). Smart nanoparticles containing enzymes were developed using different strategies such as decorating nanoparticle surface with specific enzymes acting over the tumor surface or a ligand of nanoparticle surface for releasing covalently linked molecules, nanoparticles loaded with prodrugs enzyme-activable, and nanoparticles with enzyme-degradable core for drug controlled release. In all cases, the effect of the environmental conditions to trigger the drug is crucial, particularly with enzymes located on the surface of the nanoparticle which could be exposed to extreme conditions such as high shear rate and acid pHs produced by the high metabolism of tumor cells (i.e., lactic, and acetic acids) that can inactivate and/or denature the biocatalyst. In the present work, NLC contains a hydrophobic potential drug, Viol, and a latent lipase that can be activated under physiological conditions by hydrolyzing the lipid structure of the nanoparticle with consequent release of the biodye with controlled kinetic. Also, active lipase could act over cancer cells’ membrane facilitating the contact of Viol within the cells and the entrance into the cytoplasm. Particularly, the amount of enzyme can be optimized to control the lipid hydrolysis and consequently change the amount of Viol release. Additionally, the NLC coated with Chi could guarantee the attachment of the NLC to the surface of the tumor cells (Ahmad et al., 2017).

The progress of 3D scaffolds made of biopolymer is a new trend for personalized medicine not only for the study of the development of cancer tumors but also to find novel strategies for cancer therapy, particularly after tumor resection and/or intensive treatments when surgeries are not advised (Augustine et al., 2021). In previous work, bacterial cellulose scaffolds containing SLN loaded with doxorubicin were successfully applied *in-vivo* to treat an orthotopic breast cancer mouse model. The results indicate a substantial decrease in tumor growth and metastasis, as well as drug amount by the treatment with a doxorubicin-NLC-loaded scaffold (Cacicedo et al., 2018).

The novel trends in biomedicine are involving the development of more complex structures for tissue engineering and repair, drug delivery, and organ fabrications. Among them, the 3D bioprinter allowed for the printing of hydrogels for the construction of 3D matrices (i.e., scaffolds) with defined and unique properties. Considering a personalized tumor therapy, the use of a 3D-bioprinter allows tailoring scaffolds with defined chemical composition and dimensions (i.e., size and thickness). In addition, the inclusion of the NLC into the 3D Chi-HPMC printed matrix will help to reduce the dispersion of the nanoparticles in the body. Also, the contact of the 3D scaffold containing the NLC-Viol with the solid tumor can be associated with a decrease in the administered drug amount which reduces the undesirable side effects of cancer drugs and increases patient comfort.

Finally, a novel Stimuli-Responsive Controlled Release System platform based on a 3D bioprinter and NLC containing a hydrophobic molecule actively released by an enzyme was developed. The main advantages of the platform are the versatility of the system based on: 1) the development of the composition of an appropriate nanostructured lipid carrier according to the physicochemical properties of the drug; 2) the incorporation of lipase in the NLC-drug that can control the kinetics of the drug release and the amount of the drug; 3) the inclusion of the NLC-drug-enzyme in a biodegradable 3D matrix that can be tailored based on the tumor characteristics; 4) the 3D matrix containing the drug could be placed on the tumor site avoiding drug dispersion in the body and/or inactivation and/or undesirable side effects; 5) the 3D matrix containing chitosan, a mucoadhesive and cell-adhesive polymer, can increase the contact with the tumor cells; 6) the presence of lipase and chitosan in the formulation could produce a positive effect, facilitating the entrance of the drug into the cells since both components could act over cell membranes just hydrolyzing lipids and interfering with the transport cell mechanisms.

## Data Availability

The original contributions presented in the study are included in the article/Supplementary Material, further inquiries can be directed to the corresponding author.
