# Supplementary material for: Enzymatic Active Release of Violacein Present in Nanostructured Lipid Carrier by Lipase Encapsulated in 3D-Bioprinted Chitosan-Hydroxypropyl Methylcellulose Matrix With Anticancer Activity
Source: Front Chem. 2022 Jul 7;10:914126. doi: 10.3389/fchem.2022.914126 (PMC9301079; doi:10.3389/fchem.2022.914126)

SUPPLEMENTARY TABLES

Table 1S: Structured models used to compare violacein release profiles from meshes.

Model Name	Equation
**First Order model**	$\log Q_t = log\, Q_0 + \frac{k_i t}{2.303}$
**Higuchi model**	$Q_t = K_H \sqrt{t}$
**Korsmeyer-Peppas**	$\frac{Q_t}{Q_0} = K_k t^n$
**Hixon and Crowell**	$\sqrt[3]{Q_0} - \sqrt[3]{Q_t} = K_s t$
**Baker-Lonsdale**	$\frac{3}{2}\left\{1 - \left(1 - \left[\frac{Q_t}{Q_0}\right]\right)^{\frac{2}{3}}\right\} - \frac{Q_t}{Q_o} = K_b t$

Table 2S: Characterization of nanostructured lipid carriers by dynamic light scattering. Hydrodynamic diameters (D_H), polydispersity index (PdI) and Z-potential (ζ) for NLC formulation with (NLC-Viol-Lip) and without Lip (NLC-Viol).

**Samples**	**D_H (nm)**	**PdI**	ζ
NLC-Viol	154.3	0.241	-9.78
NLC-Viol-Lip	151.5	0.319	-8.08
p-value (n= 3)	0.7584	0.017	0.0342

Table 3S: Entrapment efficiency by direct and indirect method and drug cargo per mesh matrix

**Samples**	**Entrapment efficiency (%)**			**Viol/Matrix (µmol/mg)**
	**Indirect**	**Direct**	**Average** ($\pm\sigma$)	**Direct**
Mesh-Viol	97.2	90.3	93.8 ($\pm$ 3.5)	4.67 x10^{-4}
Mesh-Viol-Lip	95.8	88.8	92.3 ($\pm$ 3.5)	4.59 x10^{-4}

Note: σ, standard deviation.

Table 4S: Data obtained by ImageJ analysis on SEM images of meshes before and after drug release.

Samples	Average mesh gap area (µm^2)	Mesh gap area square root (µm)	Grey histogram standard deviation
Mesh before drug release	283,028 (n= 12)	532.0	35.45
Mesh-Viol after drug release	424,590 (n= 10)	651.6	40.13
Mesh-Viol-Lip after drug release	660,549 (n= 8)	812.7	48.25

Table 5S: Adjusted R^2 for models fitted to Viol release profiles in Mesh-Viol and Mesh-Viol-Lip

**Model** (pH= 7.4)	**R^2adj**	
	Mesh-Viol	Mesh-Viol-Lip
First Order model	0.83	0.69
Higuchi model	0.94	0.29
Korsmeyer-Peppas	**0.95**	**0.95**
Hixon and Crowell	0.72	0.42
Baker-Lonsdale	**0.95**	0.89

SUPPLEMENTARY FIGURES

Figure 1S: Kinetic of Viol release from Mesh-Viol (●), and Mesh-Viol-Lip (○) at pH= 5.0 and 37°C. Peppas model fit of Mesh-Viol (——) (Adj r2=0.90), Mesh-Viol-Lip (– – –) (Adj r2=0.87).

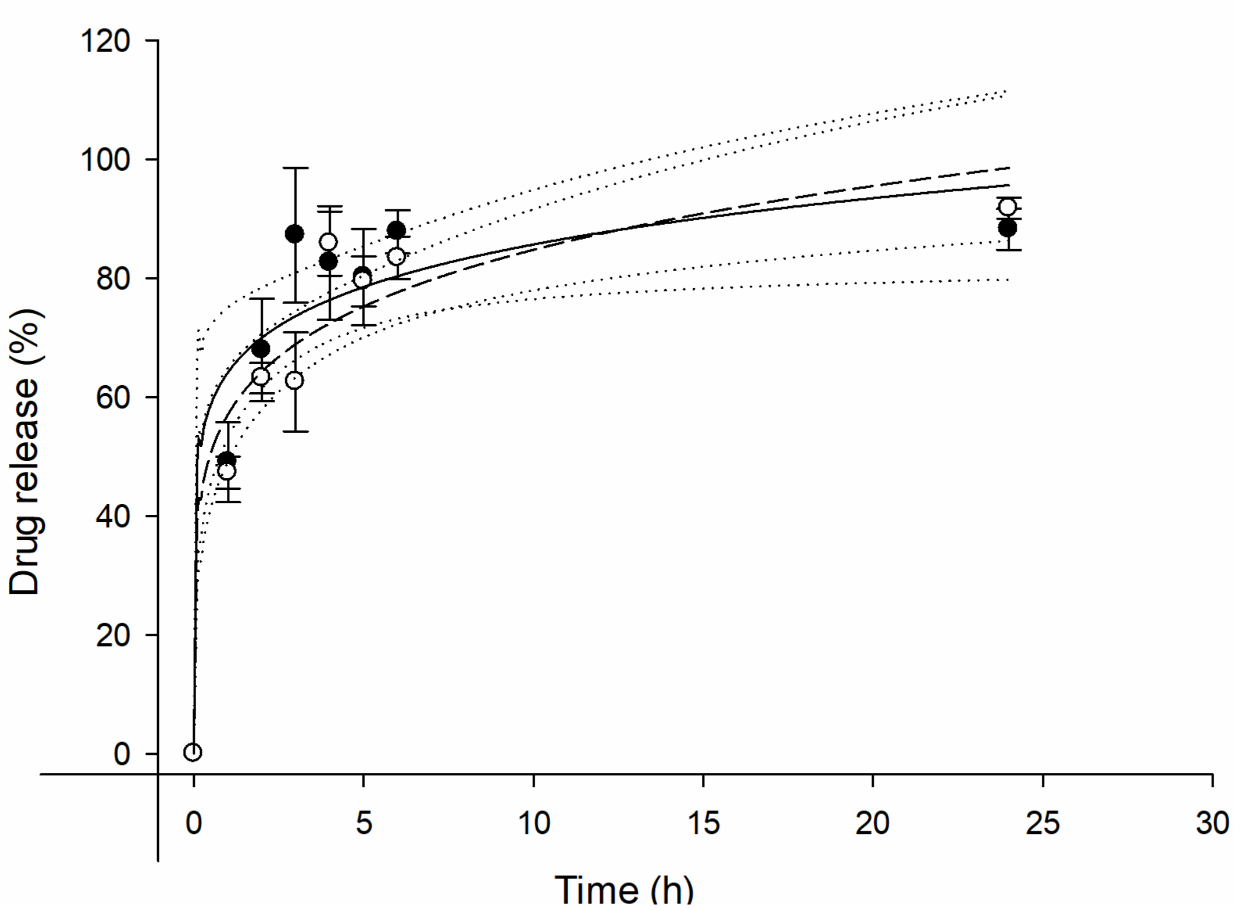

Figure 2S. Derivative of weight versus temperature for Mesh-Viol-Lip, Mesh-Viol, MM (myristyl myristate), Chi-MMW (medium molecular weight chitosan), HPMC (hydroxy propyl methyl cellulose) and P188 (Poloxamer P188). Mesh-Viol and Mesh-Viol-Lip were incubated in phosphate release buffer (pH= 7.4) for 24h before performing the analysis.

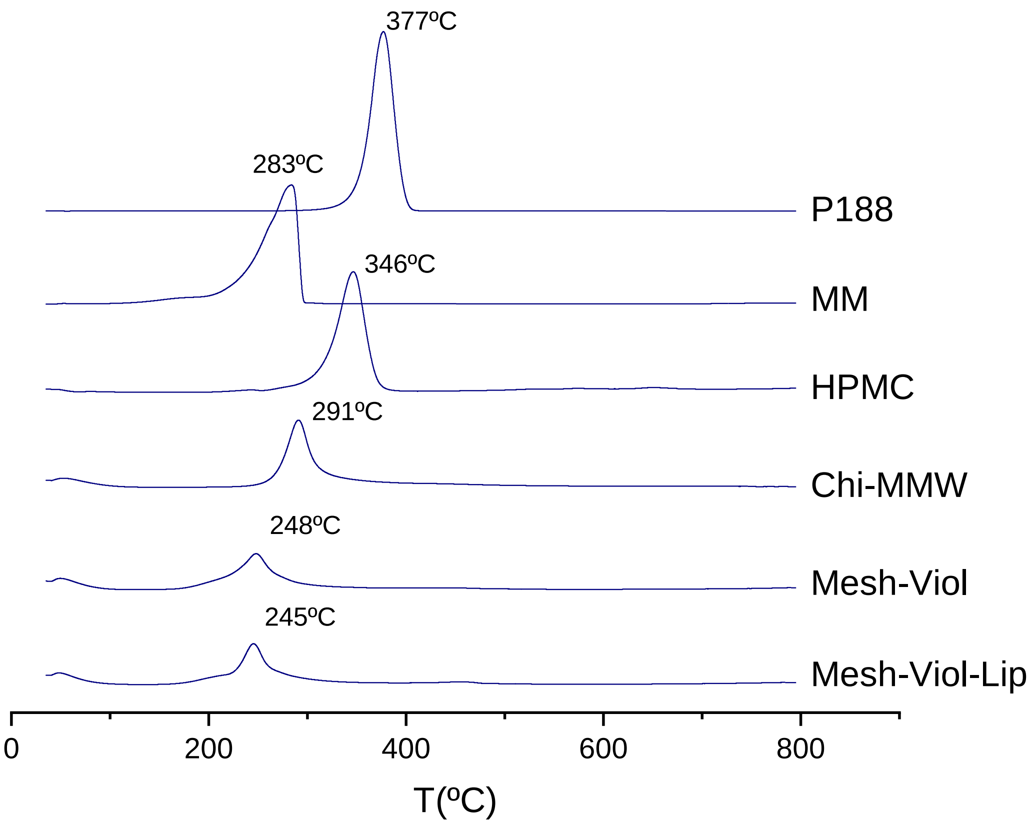

Supplement: Supplementary file 1 [file DataSheet1.PDF]
